# Supplementary material for: Quantitative electronic structure and work-function changes of liquid water induced by solute
Source: Phys Chem Chem Phys. 2021 Sep 22;24(3):1310–25. doi: 10.1039/d1cp03165a (PMC8768487; doi:10.1039/d1cp03165a)
Supplement: CP-024-D1CP03165A-s001 [file CP-024-D1CP03165A-s001.pdf]

Electronic Supplementary Material (ESI) for Physical Chemistry Chemical Physics.
This journal is © the Owner Societies 2021

Supporting Information

Quantitative electronic structure and work-function changes of liquid water induced by solute

Bruno Credidio[1,2], Michele Pugini[1], Sebastian Malerz[1], Florian Trinter[1,3], Uwe Hergenhahn[1], Iain Wilkinson,[4] Stephan Thürmer[5]*, and Bernd Winter[1]*

[1] *Molecular Physics Department, Fritz-Haber-Institut der Max-Planck-Gesellschaft, Faradayweg 4-6, 14195 Berlin, Germany*

[2]*Institute for Chemical Sciences and Engineering (ISIC), École Polytechnique Fédérale de Lausanne (EPFL), 1015 Lausanne, Switzerland*

[3] *Institut für Kernphysik, Goethe-Universität, Max-von-Laue-Straße 1, 60438 Frankfurt am Main, Germany*

[4]*Department of Locally-Sensitive & Time-Resolved Spectroscopy, Helmholtz-Zentrum Berlin für Materialien und Energie, Hahn-Meitner-Platz 1, 14109 Berlin, Germany*

[5]*Department of Chemistry, Graduate School of Science, Kyoto University, Kitashirakawa-Oiwakecho, Sakyo-Ku, Kyoto 606-8502, Japan*

Figures

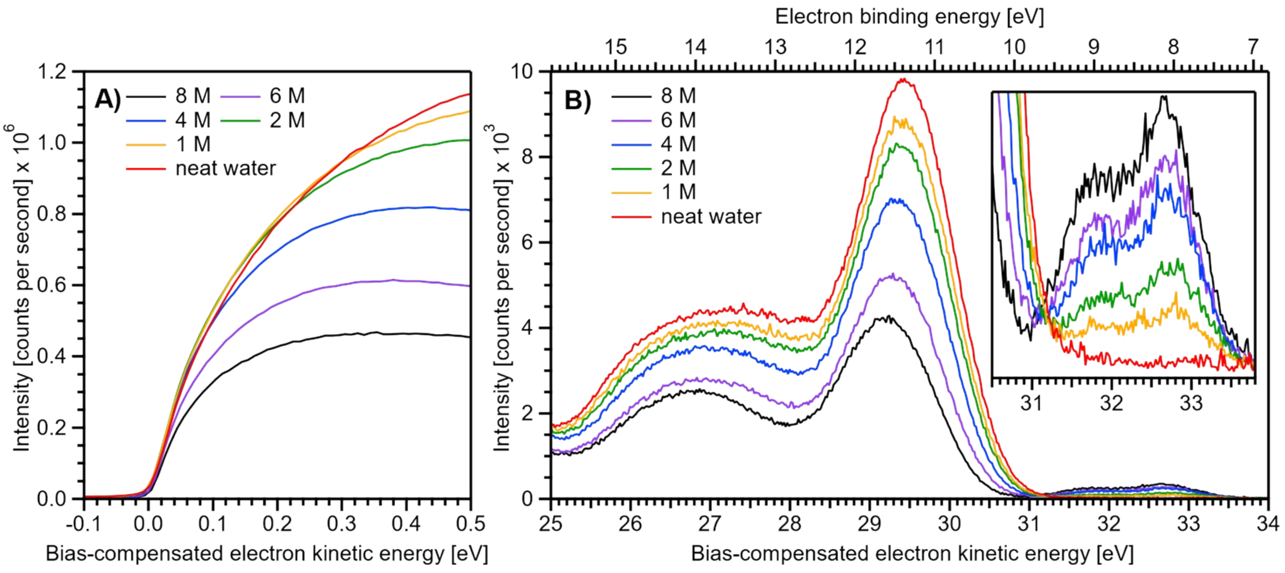

Figure SI-1: The same data for $NaI_{(aq)}$ as in Fig. 1, but here the intensity is shown as measured. The water signal decreases with higher NaI concentration.

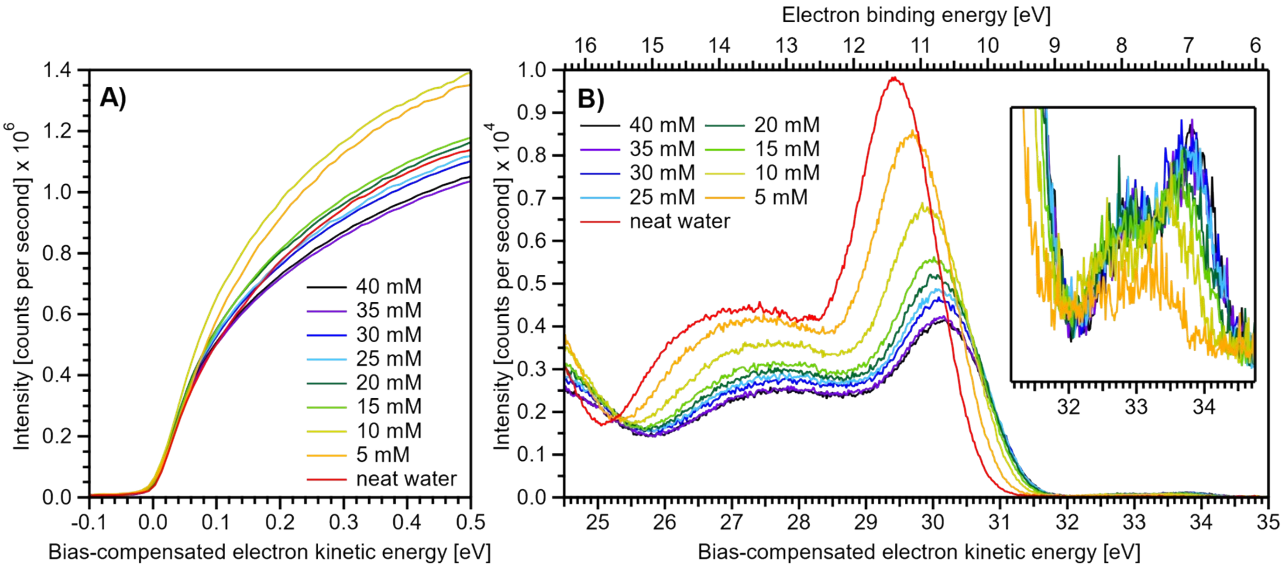

Figure SI-2: The same data for $TBAI_{(aq)}$ as shown in Fig. 5, but here the intensity is shown as measured. Similar to NaI, the water signal decreases with higher TBAI concentration.

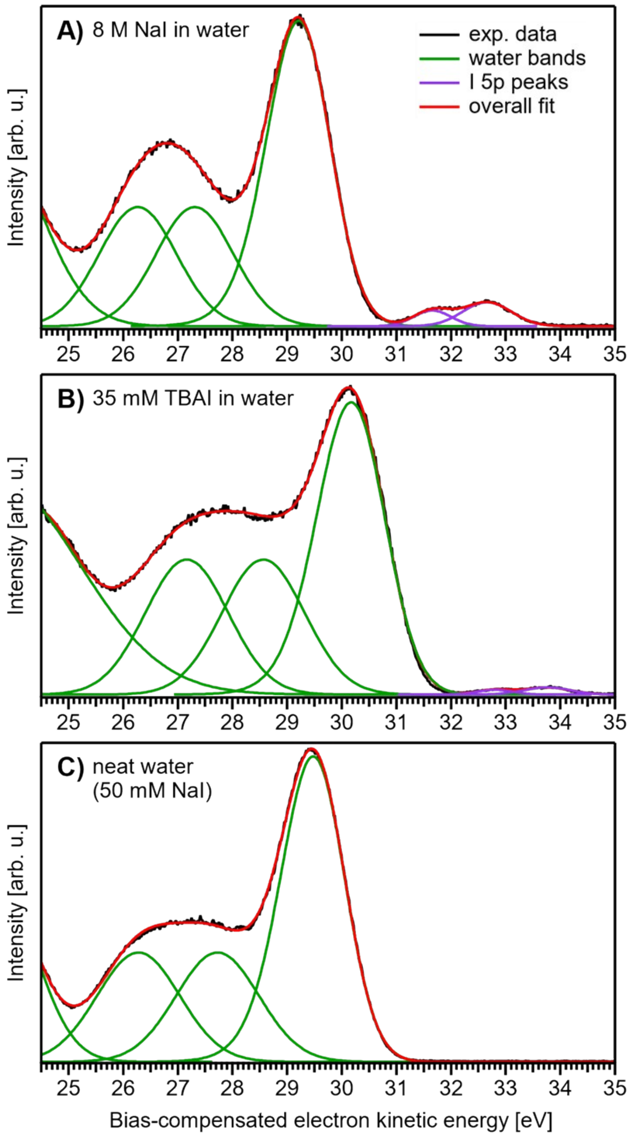

Figure SI-3: Exemplary fits to the spectra: **A)** 8 M NaI solution, **B)** 35 mM TBAI solution, and **C)** neat water (*i.e.*, with only 50 mM NaI added for charge compensation and to enable sample biasing); measured data in black, the overall fit in red, water-band features in green, and the I^- 5p doublet peak in violet. See text for details.

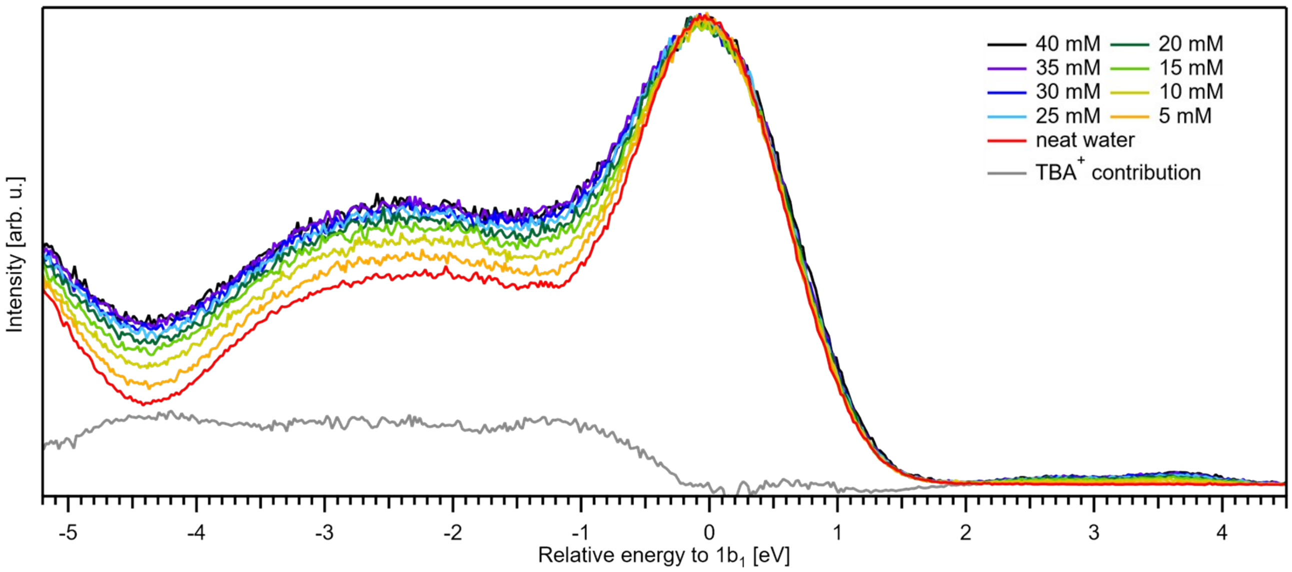

Figure SI-4: The same data for $TBAI_{(aq)}$ as in Figs. 5B and SI-2B but aligned to the same $1b_1$ peak position for better comparison of spectral changes with increasing concentration. The grey curve shows the difference between the 35-mM $TBAI_{(aq)}$ and neat water spectrum, which we assign to TBA^+.
